# Duration of Dual Antiplatelet Therapy After Stent Implantation, Still an Enigma: A Systematic Review of Randomized Clinical Trials

**DOI:** 10.7759/cureus.19549

**Published:** 2021-11-13

**Authors:** Muhammad Bin Aslam Zahid, Marrium S Memon, Mamatha Tappiti, Vignarth Shantha Kumar, Armaan M Nazir, Bhavya Koganti, Kunal Gupta, Jihan A Mostafa

**Affiliations:** 1 Department of Internal Medicine, Islamabad Medicure Hospital, Islamabad, PAK; 2 Forensic Medicine, Heavy Industries Taxila Education City (HITEC) Institute of Medical Sciences (IMS), Islamabad, PAK; 3 Department of Research, California Institute of Behavioral Neurosciences & Psychology, Fairfield, USA; 4 Deaptment of Research, California Institute of Behavioral Neurosciences & Psychology, Fairfield, USA; 5 Psychiatry, California Institute of Behavioral Neurosciences & Psychology, Fairfield, USA

**Keywords:** : acute coronary syndrome, acute myocardial infraction, stent graft thrombosis, dual anti-platelet therapy, primary percutaneous coronary intervention (pci), drug eluting stent

## Abstract

Dual antiplatelet therapy (DAPT) is used in patients after drug-eluting stent (DES) implantation to prevent stent thrombosis and ischemic events. The ideal duration of DAPT in patients after DES implantation is a topic of debate among clinicians. In the past, many research studies were published related to an optimal duration of DAPT after DES implantation.

In common practice, DAPT should be continued for one year or more after percutaneous coronary intervention (PCI) followed by DES implantation. The duration of DAPT is significant as long-term DAPT has beneficial effects but is associated with side effects like bleeding. On the other hand, short-term DAPT has a lower risk of bleeding, but it increases the rate of stent thrombosis or ischemic events. Our aim in this systematic review is to solve the dispute regarding the duration of DAPT after DES implantation. So, we tried to find the efficacy and safety of short-term (six months) DAPT by compiling data from randomized control trials (RCTs).

We conducted this systematic review following the guidelines defined in the preferred reporting items for systematic reviews and meta-analyses (PRISMA) checklist. We searched for our data from multiple databases like PubMed, Web of Science, ScienceDirect, and Google Scholar. We reviewed 10964 studies and then applied inclusion/exclusion criteria and PRISMA guidelines. Finally, we were left with only 21 studies regarding the optimal duration of DAPT after DES implantation. Our systematic review will help determine the non-inferiority of short-term (six months) DAPT to long-term (12 months) DAPT. Furthermore, we also noticed with short-term (six months) DAPT, there was decreased incidence of bleeding as compared to DAPT for long-term. But more studies were required to establish the safety and effectiveness of short-term (six months) DAPT compared to long-term (12 months) DAPT in patients after DES implantation.

## Introduction and background

Around $219 billion in 2014 and 2015 was spent in the United States on heart diseases, including pharmaceutical and health care services related to heart disorders [1]. According to figures from the American Cardiology Association, 365,914 people were diagnosed with coronary heart disease in 2017 [2]. Dual antiplatelet therapy (DAPT) is prescribed to 1.2 million patients each year following drug-eluting stent (DES) implantation. For treating thrombotic stroke, coronary artery disease (CAD), peripheral vascular disorders, and transient ischemic attack (TIA), DAPT is commonly prescribed in more than seven million patients globally [3].

Drug-eluting stent (DES) implantation after percutaneous coronary intervention (PCI) is the management of choice in a patient with the acute coronary syndrome (ACS) and stable angina. However, in the months following DES implantation, both aspirin and an additional platelet receptor (P2Y12) inhibitor are required to reduce the risk of stent thrombosis [4]. Extended DAPT use lowers the risk of ischemic events but raises the risk of bleeding events. As a result, there is a critical trade-off between the dangers of ischemia and bleeding that must be carefully considered when determining the duration of DAPT [5].

Platelets are those tiny particles present in the blood responsible for the formation of blood clots, and they are the main reason for a sudden blockage of a coronary stent and a heart attack. The antiplatelet agent is a class of drugs that prevents platelet from clumping together and forming blood clots. Many patients with heart problems and stroke are prescribed two types of antiplatelet agents to prevent clotting. When two types of antiplatelet drugs are taken together, they are very effective and efficient in preventing blood clots. This is called dual antiplatelet therapy [6]. Dual Antiplatelet therapy consists of aspirin and, in addition to that second type of antiplatelet agent, called a P2Y12 receptor inhibitor (Clopedogrel), which is usually prescribed in combination with aspirin for months or years after PCI with DES implantation.

Subsequently, it is a class one recommendation that DAPT continues for a minimum of 12 months after revascularization and ischemic heart disease. Surprisingly, the European Society of Cardiology (ESC) and American Heart Association (AHA) guidelines regarding the duration of DAPT after DES implantation differ, ESC suggesting six to 12 months, and the AHA recommends at least 12 months following DES implantation in the stable patient [7-9]. Both AHA and ECS recommend at least 12 months of DAPT in patients with acute coronary syndromes (ACS), with the possibility of extending the duration beyond 12 months in patients with a low risk of bleeding and six months in patients with a high risk of bleeding [9].

There is a significant risk of bleeding after DAPT for 12-months or more in high-risk patients, and there are more chances of stent thrombosis following DES implantation with DAPT for less than 12-months. Our study's primary objective or goal is to understand and identify the most effective and valuable duration of DAPT after DES implantation. The main emphasis is on the risk of an ischemic event, stent thrombosis, and bleeding risk evaluation. So, we concluded that DAPT for the short-term (six months) has the same clinical outcome as 12-month DAPT in preventing stent thrombosis following DES implantation. We can safely administer DAPT for six months after PCI and DES implantation to patients with a high likelihood of bleeding.

The optimal duration of DAPT following PCI is still up for dispute. Many previous meta-analyses of randomized controlled trials (RCTs) have sought to answer whether DAPT for 12 months is superior to DAPT for six months after implantation of DES. Most RCTs had limitations regarding statistical power; the leading cause behind this is the rarity of stent thrombosis-related events. Furthermore, the safest and shortest duration is still to be determined. There were no significant changes in antithrombotic efficacy between short-term and long-term DAPT regimens. In addition to that, extended dual antiplatelet therapy treatment induces an increased risk of bleeding [10].

We included many recent RCTs in our study, so our analysis has more statistical power. We wanted to see how effective short-term dual antiplatelet therapy is compared to long-term DAPT after DES implantation, with a particular focus on the chances of stent thrombosis with new DES.

## Review

Methodology

We did this systematic review by using preferred reporting items for systematic reviews and meta-analyses (PRISMA 2020) Figure 1.

**Figure 1:**
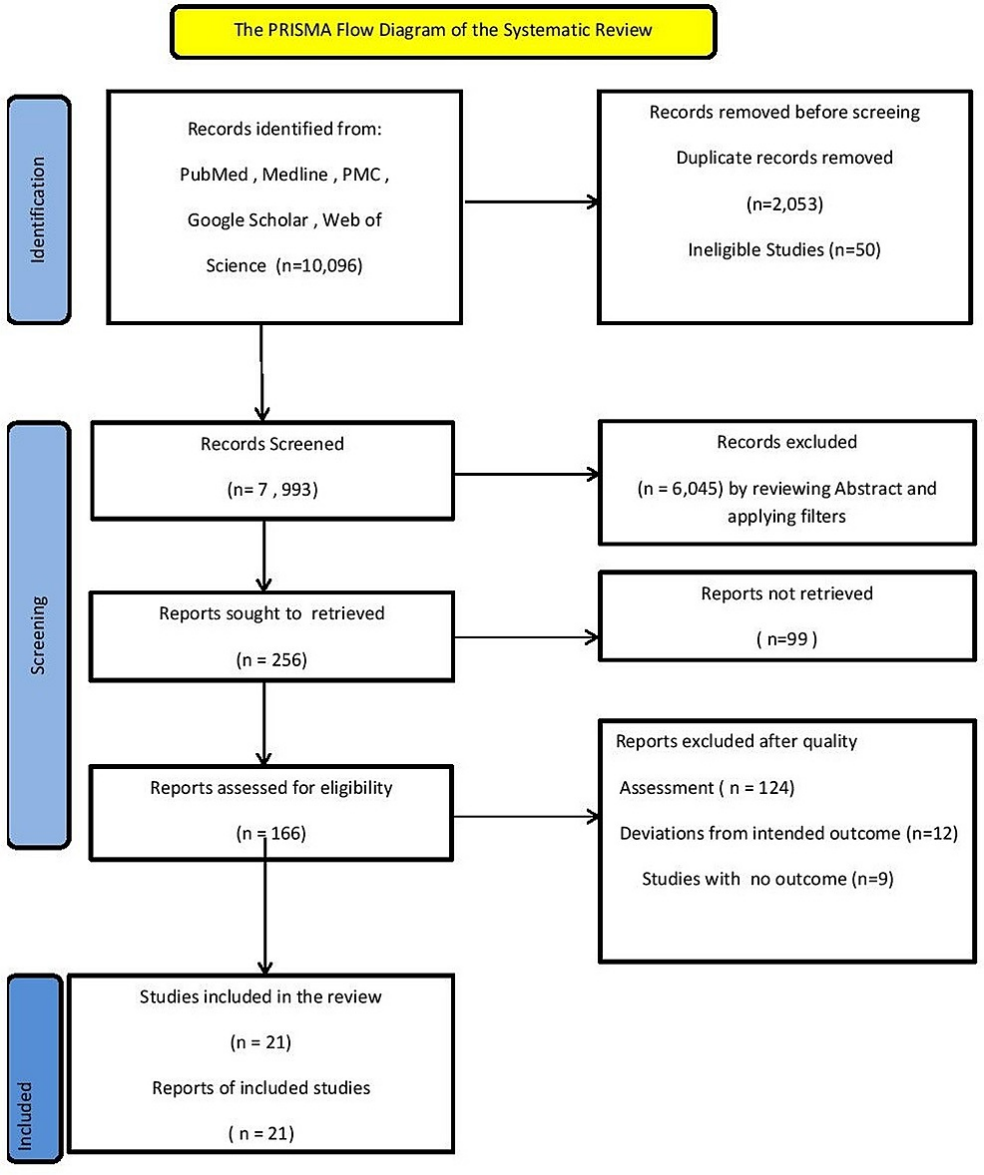
The PRISMA Flow Diagram of the Systematic Review PRISMA: preferred reporting items for systematic reviews and meta-analyses

Inclusion/Exclusion Criteria

We included studies from the last five years, i.e., 2015 to 2020, regarding the ideal period of dual antiplatelet after drug-eluting stent (DES) implantation. We selected studies done on humans and which are only in the English language. We included studies only with the patient who had DES implantation. Consequently, we excluded all studies published before 2015.

Data Source and Search Strategy

We searched for randomized clinical trials (RCTs) published between 2015 to 2020 in PubMed, Science Direct, Web of Science, and Google Scholar from 18 August 2021 to 22 August 2021. We followed MeSH and keyword strategy to search for related RCTs. We searched relevant articles by applying keywords and MeSH terms individually. We summarized MeSH terms used in Table 1 and keywords used in Table 2. 

**Table 1:** MeSH Terms used to search data

MESH TERMS USED	DATABASE USED
("Acute Coronary Syndrome/anatomy and histology"[Mesh] OR "Acute Coronary Syndrome/complications"[Mesh] OR "Acute Coronary Syndrome/drug therapy"[Mesh] OR "Acute Coronary Syndrome/therapy"	PubMed
("Dual Anti-Platelet Therapy/adverse effects"[Mesh] OR "Dual Anti-Platelet Therapy/epidemiology"[Mesh] OR "Dual Anti-Platelet Therapy/methods"[Mesh] OR "Dual Anti-Platelet Therapy/organization and administration"[Mesh] OR "Dual Anti-Platelet Therapy/pharmacology"[Mesh] OR "Dual Anti-Platelet Therapy/standards"[Mesh] OR "Dual Anti-Platelet Therapy/therapeutic use"[Mesh] OR "Dual Anti-Platelet Therapy/trends"[Mesh])	PubMed
("Drug-Eluting Stents/classification"[Mesh] OR "Drug-Eluting Stents/history"[Mesh] OR "Drug-Eluting Stents/organization and administration"[Mesh] OR "Drug-Eluting Stents/pharmacology"[Mesh] OR "Drug-Eluting Stents/therapeutic use"[Mesh] OR "Drug-Eluting Stents/trends"[Mesh])	PubMed

**Table 2:** Keywords used to search data

KEYWORDS	DATABASE USED
Coronary stenosis OR Myocardial infraction OR heart disease	PubMed
Antiplatelet agent OR antiplatelet aggregation OR blood thinner OR duration	PubMed
Stent implantation OR drug-coated stent OR bare-metal stent	PubMed

Data Extraction

The randomized controlled trials (RCTs) we selected were screened separately by two reviewers. We have extracted sample size, study outcome, study design, and year of publication. In case of conflict, we solved them with mutual discussion on the study in question.

Quality Assessment

As we included randomized controlled trials, so we used the following tool for quality assessment: Cochrane bias assessment.

Result

We used PubMed, Science Direct, Web of Science, and Google Scholar to search relevant articles. Initially, our search yielded 1,0962 results. We removed duplicates, and we were left with 2,053, and 50 studies were ineligible. Then we screened and read titles and abstracts thoroughly, and we applied inclusion and exclusion criteria. In the end, we included 21 studies in our systematic review.

Discussion

Dual antiplatelet therapy (DAPT) is the most vital part of treatment after percutaneous coronary intervention (PCI) followed by drug-eluting stent (DES) implantation, so the appropriate duration should be standardized. So that, patients had more benefits like decreased chances of the ischemic event without having significant side effects.

Dual Antiplatelet Therapy for 12 Months or More After PCI

In 2015 randomized clinical trial (RCT) study was published, which included 11,962 participants. In addition to that, this trial included 30% of the participants who had a myocardial infarction (MI). This study data showed that the participants who presented with MI and had DES implantation benefited from continued DATP beyond one year by decreasing the chance of stent thrombosis. Furthermore, continuing clopidogrel alone for 18 months in patients with MI and without MI was associated with an absolute risk reduction of MI and for stent thrombosis [11]. As with the decreased risk of ischemic events, there were increased chances of bleeding with an extended duration of DAPT. The patients at risk of the ischemic event were also at high risk for the bleeding event, so the result extracted from this study was not beneficial for those at risk of bleeding. This study was an exception to all other trials, which favors the short-term (six months or less) DAPT non-inferiority to long-term (12 months or more) DAPT in patients with or with MI.

Another RCT named (SMART-DATE) in 2018 was a randomized non-inferiority trial. They enrolled the patient who had STMI or unstable angina in that trial. In this trial, the primary endpoint or the event rate is 4.7% (63 patients) of participants who had DAPT for six months vs. 4.2% (56 patients) in patients who had DAPT for 12 months. So there is an absolute risk of .5 % of the ischemic event in patients who had DAPT for six months or short-term, so non-inferiority of six months (short-term) DAPT was not proved [12]. This trial established increased chances of MI with six months of DAPT. Subsequently, DAPT for 12 months or more remains standard care without risk of excessive bleeding. This RCT enrolled 2712 patients with prior MI and acute coronary syndrome (ACS) and concluded an increased risk of MI in the patient who had DAPT for six months or shorter duration. So, the safety and efficacy of short-term (six months or less) DAPT were not established in the patients with ACS or patients with prior MI. And the extended period (12 months or more) of DAPT in ACS patients without excessive bleeding remains the best option [12].

According to the conclusion of both trials, the benefits of long-term DAPT outweigh the risk of bleeding. However, both RCTs included a large number (n=13967) of participants and have high power and authenticity. But both studies only included the participant who remained event-free after PCI for 12 months and was complaint to DAPT. Moreover, most of the participants were not randomized according to stent and drug type. Both the studies were an exception to all other trials, which favors the short-term DAPT non-inferiority [13-15] to long-term DAPT in patients with or with MI. The studies that favor non-inferiority had randomization according to drug and stent type, which reduced the margin of bias.

Dual Antiplatelet Therapy for Six Months or Less After PCI

Two RCTs, ISAR SAFE [16] and ITALIC [17], in which a total (n=5058) participants enrolled. 2921 were randomized to six months DAPT therapy, and 2103 were randomized to long-term DAPT therapy. By considering the clinical outcome of both trials, the non-inferiority was established of short-term DAPT (six months or less) to DAPT for long term (12 months or more) in both studies. The primary endpoints of clinical outcome are a composite of death, myocardial infarction, stent thrombosis, stroke, and significant bleeding. There is no significant difference in the clinical outcome regarding the primary endpoints in both studies with short-term (six months) DAPT.

In OPTIDUAL [18] trial, 1799 patients were enrolled after DES implantation. This study's findings were consistent with older trails conclusions like ISAR SAFE [16] and ITALIC [17] and did not prove the superiority of extended DAPT to short-term DAPT. So, according to this study, extended or more than 12 months DAPT had no statistical superiority to short-term DAPT in terms of clinical outcome or prevention of ischemic event [14].

Another RCT concluded non-inferiority of six-month therapy. However, in this study, there is no increase in the risk of bleeding with extended DAPT. But this finding can be due to the reason that participants enrolled for extended DAPT have already tolerated DAPT for six months. The majority of bleeding episodes occurred in the initial six months after starting DAPT. This study also endorsed the safety and efficacy of six-month or short-term DPT after new DES [15].

The ITALIC trial concluded that chances of bleeding and stent thrombosis at one year were the same with 12 months vs. six months of DAPT after PCI with DES implantation. There were 2031 patients included in this trial. This trial is better because it also addresses the safety and efficacy of short-term DAPT after PCI in stable patients and patients with ACS. The analysis according to ITALIC subgroups showed no difference among short-term and long DAPT. However, patients with prior MI who had extended DAPT to some extent have lower chances of complications [17].

DAPT-STEMI [18] trial on 1100 patients contradicts the 2017 ITALIC trial regarding patients with prior MI benefits with extended DAPT. This trial established that six-month therapy is non-inferior to 12 months in patients even after ST-elevation myocardial infarction (STMI), which is a fundamental inconsistency to studies with a more significant number of participants suggested that after STMI, long-term dual antiplatelet therapy is more beneficial in preventing stent thrombosis or ischemic events. So, this trial is the first of its type, proving the safety of short-term DAPT in a patient with ST-elevation MI after DES implantation. RCT study published in 2020 further endorsed that the short-term duration of the DATP has the same efficacy in patients with ACS and stable coronary artery disease.

The recent study STOP-DAPT was done in which 3045 participants were included, and 2974 completed the trials. In older (RTCs), the studies focus on six months of DAPT in the patients after stent implantation. But this trial took one step ahead and focused on efficacy and safety of one-month DAPT followed by clopidogrel in the patients, which is a bold step as there are some old studies still of the view that after ACS, the standard treatment should be 12 months or more DAPT [19]. This trial concluded DAPT for one month followed by clopidogrel decreases the risk of ischemic and bleeding events. In that way, it favors non-inferiority and non-superiority of one-month DAPT to 12-month DAPT.

All the trials mentioned above have findings contrary to the clinical trial conducted, with more participants (n= 11,962) [11]. The data from this study suggested that long-term DPAT is favorable in patients who have stent implantation after MI regardless of the type of stent. But there were many trials conducted and published from 2015 to 2020, which proved the non-inferiority of six months DAPT to 12-month DAPT.

One RCT done on 25,682 subjects also supported that extended DAPT increases the risk of bleeding [20]. This RCT was a placebo control trial that was double-blinded and randomized that compared the short- and long-term duration of DAPT after DES implantation. This study showed an increased risk of bleeding with continuing DAPT more than 12 months after stent implantation. Interestingly, this study has been an out-of-the-way example of finding the relation between mortality and continued DAPT. We extracted some significant conclusions from this trial.

Most importantly, the DAPT beyond one year has been beneficial in preventing stent thrombosis in patients with MI, but there has been an increased risk for bleeding. As in this study, the stent type was not randomized, so data suggest increased non-cardiovascular mortality for DES and less for bare-metal stunts. It happened because there were large numbers of cancer patients who had DES. This RCT is an exception to other trials as it focuses on the effects of long-term DAPT treatment on randomized patients who had everolimus-eluting stent implantation.

All the studies mentioned above proved that short-term DAPT has a similar clinical outcome as that of long-term DAPT. Some of the studies included in our study are summarized in Table 3.

**Table 3:** Some studies regarding the Dual Antiplatelet Therapy included in our study The table shows the studies on the duration of dual antiplatelet therapy, after PCI majority of studies proved non-inferiority of six-month DAPT to long-term DAPT. DAPT: dual antiplatelet therapy, Long-Term DAPT: dual antiplatelet therapy for 12 months or more than 12 months, PCI: percutaneous coronary intervention

Author	Participants Included	Name of Study	Hypothesis	Result
Hahn et al., 2015 [12]	2,712	SMART -DATE	Six-months DAPT non-inferior to long-term DAPT	NOT PROVED
Han et al., 2016 [13]	1,850	I-LOVE-IT 2	Six-months DAPT non-inferior to long-term DAPT	PROVED
Helft et al., 2016 [14]	1,385	OPTIDUAL	The superiority of long-term DAPT (STOPED)	NOT PROVED
Schulz et al., 2015 [16]	4,000	ISAR-SAFE	Six-months DAPT non-inferior to long-term DAPT	PROVED
Didier et al., 2017 [17]	1,850	ITALIC	Six-months DAPT non-inferior to long-term DAPT	PROVED
Kedhi et al., 2019 [18]	1,100	DAPT-STEMI	Six-months DAPT non-inferior to long-term DAPT	PROVED
Watanabe et al., 2019 [19]	2,974	STOP DAPT	One-month DAPT was both non-inferior and non-superior	PROVED
Hong et al., 2016 [21]	1,400	IVUS-XP	Six-months DAPT non-inferior to long-term DAPT	PROVED

In addition to that, there is an increased risk of bleeding with long-term DAPT [11]. This study included 25,685 participants, which has significant power and proved an increased risk of bleeding with extended duration DAPT. Studies that proved the non-inferiorityod of short-term DAPT to long-term DAPT had included many patients, so the risk of bias is very minimal. These studies were better and had less chance of bias because the participants were randomized according to the stent and drug type in most of the studies.

A study recently in 2020 proved non-inferiority of six months to 12 months also included the patients with ACS and MI and addressed other risk factors for ischemic heart disease, which makes this study more authentic and less chance of bias.

Different Types of Stents and Duration of Dual Antiplatelet Therapy

There was an RCT conducted in which a total of 1,400 patients (implanted mean total stent length >45 mm) were assigned to receive six-month or 12-month DAPT [21]. The noticeable feature of this study was that it included the patients with long-length DES, which was not considered in older studies. Older studies usually had a median stent length for stents and different types of stents. In older clinical trials, the type of stent before enrolling the participants was not randomized. That's why the question that arises is if there is any specific type of stent after which a specific duration of DAPT will be more favorable? A recent RCT enrolled the patients with everolimus-eluting stents (EES) implantation. This trial depicted that short-term DPAT has an overall low risk of stent thrombosis and a low rate of bleeding when we use EES [21]. There was another trial named I LOVE IT [12] in which they included the patients with biodegradable polymer - DES. In the past, several RCTs were conducted to prove the safety and efficacy of short-term DAPT in a patient with different stents. This RCT [13] enrolled 1829 patients, who had biodegradable polymer sirolimus-eluting stents. Its results are consistent with other previous trials, which included patients with drug-eluting stents. These trial results suggested that short-term DAPT had the same efficacy and safety as long-term DAPT if we use DES. So it means that if we use more contemporary DES, then the duration of DAPT can be tailored according to patient need and risk factors safely [15].

Our analysis favors this finding that with appropriate second-generation DES implantation, short-term DAPT is non-inferior to long-term DAPT. In addition to that, there is less risk of bleeding along with a decreased risk of stent thrombosis.

Limitations

There are a few flaws in this study. For example, we only focused on clopidogrel-based DAPT duration; thus, the results may be different if you use alternative P2Y12 inhibitors like prasugrel or ticagrelor. Furthermore, several RCTs did not report endpoints. The type of stents was also not adequately randomized in some RCTs.

The data we collected from the English-language publications limit additional data on the optimal duration of DAPT, excluding some crucial data regarding DAPT optimal duration after PCI and drug-eluting stent implantation. Another limitation in this study is the inclusion of data reported between 2015 and 2020, excluding vital data published before 2015.

## Conclusions

Dual antiplatelet therapy (DAPT) is the central core treatment after percutaneous coronary intervention (PCI) to prevent ischemic events. In our analysis, we have tried to determine the ideal duration of DAPT by compiling the data from randomized controlled trials (RCTs). We observed that short-term DAPT has equal efficacy as that of the standard period of DAPT. We tried to improve the power and authenticity of our study by including only RCTs published in the last five years regarding the duration of DAPT after drug-eluting stent (DES) implantation. It is one step forward to establish an ideal or patient-tailored approach to increase the benefits of DAPT, which outweighs the side effect of DAPT like bleeding. Our analysis will have very positive implications for the students and pave the way for students to dig more deeply into the benefits or efficacy of short-term DAPT. Short-term DAPT will also improve patient compliance towards DAPT, as it is tough for some patients to comply with prolonged drug therapy. This study will ease the clinicians in deciding the regime for the high-risk patients who underwent stent implantation. A future recommendation in cardiology is conducting more studies to investigate further the efficacy of short-term DAPT to avoid the side effects of long-term DAPT therapy. Moreover, the ideal duration of DATP after PCI and stent implantation is still up for debate, and there is still a need for further studies to solve this enigma.
